# Ventriculoperitoneal Shunt Infections: Causative Pathogens and Associated Outcomes from Multiple Hospitals in Saudi Arabia

**DOI:** 10.3390/jcm14062006

**Published:** 2025-03-16

**Authors:** Mohammed Alqasmi, Yousif A. Kariri, Rawaf Alenazy, Mohammed Thabet, Ghaith Fallata, Nasser Alqurainy

**Affiliations:** 1 Department of Medical Laboratory Sciences, College of Applied Medical Sciences-Shaqra, Shaqra University, Shaqra 11961, Saudi Arabia; 2 Department of Biology, Faculty of Science, Al-Baha University, Al-Baha 65779, Saudi Arabia; 3 Department of Basic Science, College of Science and Health Professions, King Saud bin Abdulaziz University for Health Sciences, Jeddah 22384, Saudi Arabia; 4 Infectious Disease Research Department, King Abdullah International Medical Research Center, Jeddah 22384, Saudi Arabia; 5 Ministry of the National Guard-Health Affairs, Riyadh 11426, Saudi Arabia; 6 Department of Basic Science, College of Science and Health Professions, King Saud bin Abdulaziz University for Health Sciences, Riyadh 11426, Saudi Arabia; 7 Infectious Disease Research Department, King Abdullah International Medical Research Center, Riyadh 11426, Saudi Arabia

**Keywords:** hydrocephalus, ventriculoperitoneal shunt, hospital-acquired infections, infections, pathogens, multidrug resistant, clinical outcomes, Saudi Arabia

## Abstract

**Background:** Ventriculoperitoneal shunting (VPS) is the primary treatment for hydrocephalus, significantly improving patients’ outcomes. However, it is marred by high failure rates due to infections, which account for a third of these malfunctions and escalate morbidity, mortality, and healthcare costs. **Method:** This study focused on evaluating VPS infection rates, pathogens, their resistance patterns, and the impact on clinical outcomes using retrospective data from multiple hospitals in Saudi Arabia. It included data from hydrocephalus patients who underwent VPS and only considered positive cultures that were confirmed from CSF or shunt tip samples. **Results:** This study excluded patients with prior infections before VPS placement or those treated with alternatives to VPS. Out of 317 patients who met the inclusion criteria, the analysis revealed that 23 patients (7.26%) suffered from VPS infections, mostly within the first two weeks post-surgery (58.06% of cases), with a significant discrepancy in infection rates between hospitals. Infections predominantly involved Gram-positive bacteria (58.08%), especially coagulase-negative *staphylococci* and *Staphylococcus aureus* (25.81% and 12.90%, respectively). There was also a substantial presence of Gram-negative bacteria and fungi, accounting for 35.46% and 6.46%, respectively. Despite general antibiotic susceptibility, resistance was significant in certain cases, including multidrug-resistant isolates like *Klebsiella pneumoniae*, *Pseudomonas aeruginosa*, and *Acinetobacter ursingii*. Importantly, patients with VPS infections had a tenfold increase in the length of hospital stay (70.84 days, SD ± 139.5) compared to non-infected patients (7.69 days, SD ± 20.72), indicating high morbidity and associated treatment costs. **Conclusions:** Our results emphasize the importance of better VPS infection control and standardized hospital protocols to decrease the incidence of VPS-related infections, both in Saudi Arabia and globally.

## 1. Introduction

Hydrocephalus is a medical condition characterized by the accumulation of cerebrospinal fluid (CSF) within the ventricles of the brain [1]. This life-threatening condition leads to dilated cerebral ventricles, a thinned cerebral mantle, and increased intracranial pressure [1,2]. There are several etiological factors of hydrocephalus, which can be either congenital anomalies, genetic diseases, or medically acquired conditions. The latter can result from multiple causes, such as brain tumors, infections, or hemorrhages. However, a significant proportion of patients develop the disease with unknown cause [3,4]. Globally, an estimated 80 to 125 infants per 100,000 births are diagnosed with congenital and acquired hydrocephalus, with variations by region [5].

Hydrocephalus is typically treated with one of two surgical options: ventriculoperitoneal shunt (VPS) or endoscopic third ventriculostomy (ETV). While VPS is used for both obstructive and communicating hydrocephalus across all age groups, ETV is limited to obstructive conditions and is often less successful in infants [3,6]. For that reason, VPS is the most commonly used approach for treating hydrocephalus [7]. It exudes excess CSF by a ventricular catheter (shunt) inserted into the brain and connected to a valve controlling the distal catheter. The latter catheter is placed in the peritoneal cavity for CSF drainage [8]. However, while VPS improves life expectancy and neurological function, shunt malfunctioning can occur, resulting in multiple complications to the patients [9,10]. Infection is the second most common cause of VPS malfunction, following physical damage to the device, and is the primary reason of early shunt failure, posing a life-threatening risk to patients [9,11,12].

VPS infections are common, affecting 5–15% of patients, with a majority of infections developing a short time after shunt placement [13,14,15]. VPS infections are typically caused by the skin flora, predominately opportunistic staphylococci [16]. Coagulase-negative *staphylococci* (CoNS) are the most reported microorganisms, and among them, *Staphylococcus epidermidis* is the most common, accounting for over half of VPS infection cases [16,17,18]. *Staphylococcus aureus* is another leading cause of VPS infection, accounting for up to 25% of cases, followed by a number of Gram-negative pathogens such as *Enterobacteriaceae* and pathogenic non-fermenting bacteria [16,17]. A common trait among these pathogens is their ability to form biofilms, which protect them from the host’s immune system and antibiotic treatments. For that reason, shunt removal and sterilization of the CSF using antibiotics are the two major treatment interventions to tackle VPS infections, although the treatment can become more complicated when a multidrug-resistant (MDR) pathogen is involved [19,20,21].

VPS infections significantly challenge clinical settings, increasing morbidity and mortality while straining healthcare resources. Individuals recovering from VPS infections often experience reduced intelligence levels, more frequent seizures, and lower overall quality of life [22]. Moreover, these complications impose a significant financial burden on healthcare systems, with annual treatment costs for shunt infections estimated at USD 50,000 per patient [22,23]. 

Despite the availability of global data, there is a notable gap in comprehensive research on VPS infections in the Middle East, particularly in Saudi Arabia. This region presents unique challenges and demographics that are currently under-represented in the literature. Therefore, this study aims to address this gap by gathering and analyzing data from multiple hospitals in Saudi Arabia to determine the prevalence of VPS infections and identify the causative organisms and their antibiotic resistance profiles. Additionally, this study’s objectives extend to assessing the clinical outcomes of VPS infections in patients by measuring the length of hospital stay (LOS) as a primary indicator of morbidity. In doing so, it seeks to optimize the management of VPS-associated infections and elucidate the variability in treatment strategies across different hospital settings.

## 2. Materials and Methods

### 2.1. Research Setting and Study Population

Data from patients with hydrocephalus who underwent VPS placement in multiple hospitals were retrospectively reviewed to determine infection rates, causative pathogens along with their antibiotic resistance profiles, and associated clinical outcomes. The King Abdullah International Medical Research Center (KAIMRC) collected the data from various hospitals operated by the Ministry of National Guard Health Affairs (MNGHA) in Saudi Arabia, which collectively have over 3700 beds (Table 1). The inclusion criteria included patients with a diagnosis of hydrocephalus who had undergone VPS placement. For infection cases associated with VPS, only CSF samples or shunt tip cultures showing microbial growth post-VPS placement were considered. The exclusion criteria included patients treated with methods other than VPS. Moreover, preoperative infections were rigorously reviewed, and any infections recorded before the date of VPS placement were excluded to ensure they did not confound the postoperative infection data and to avoid documenting pre-existing infections. Additionally, to prevent data redundancy, microbiology results from follow-up examinations of primary infections were also excluded. Cultures from follow-up examinations were recognized in our study as those collected on consecutive days after the initial positive culture. This approach was necessary because routine monitoring by physicians, which is aimed at assessing infection status, could inflate prevalence rates by repeatedly documenting the same organism in laboratory reports.

### 2.2. Data Collection and Analysis

Data were retrospectively reviewed for the period from January 2021. The data were initially recruited and managed by the KAIMRC before being securely transferred to the study investigators for subsequent data extraction and analytical processing. The dataset primarily included patient demographics, underlying conditions, surgical procedures, microbiology lab findings, and lengths of hospital stay.

The dataset was exported into a Microsoft Excel (version 16.89.1)-compatible format for computer-based analysis, followed by a thorough review to ensure accuracy. Patient data were manually revised and extracted according to this study’s inclusion criteria. Statistical analyses were conducted using GraphPad Prism (version 6.01), focusing on descriptive statistics, including the calculation of means, standard deviations, medians, and frequencies. Moreover, one-way ANOVA followed by Dunnett’s multiple comparison test was used to identify significant differences in the length of hospital stay (LOS) among patient groups. A *p*-value below 0.05 was considered to indicate statistical significance.

### 2.3. Ethical Approval

Ethical approval for the study design and data collection was granted by the Institutional Review Board (IRB) at KAIMRC (H-01-R-005) (ref.#: RYD-23-419812-157726, 15 October 2023), in accordance with the Declaration of Helsinki. To ensure participant privacy and confidentiality, no personal information was requested, and patient identities were anonymized.

## 3. Results

### 3.1. Demographics

A total of 317 patients diagnosed with hydrocephalus and treated with a VPS were included in the analysis (Table 2). The cohort comprised 151 males (47.63%) and 166 females (52.36%). The body mass index (BMI) of the patients showed significant variation, with the majority (52.05%) being underweight (BMI less than 18.5). A smaller proportion had a normal BMI (16.72%), while 12.93% were overweight, and 10.73% fell into obesity class I (BMI 30-34.9). The BMI for each patient was determined by averaging the BMI values from all recorded visits, ensuring an accurate representation of each patient’s BMI over the study period.

Of the recorded birth conditions, 31 patients (9.78%) were full-term and 43 (13.56%) were pre-term, while the majority did not have a specified birth condition in the database. In contrast, the age distribution at the first VPS procedure among patients indicated significant early-life interventions, with 27.44% of procedures performed in infants under 6 months. This necessity for early surgery slightly declines in late infancy, with only 3.47% of procedures occurring between 7 and 12 months. Notably, there is a notable peak in shunt interventions during childhood and adolescence (23.34% between ages 3 and 16). Adult interventions were also substantial, with 25.55% of first shunts placed between ages 17 and 65, while elderly patients (over 65 years) represented 9.78% of shunted patients (Table 2).

The distribution of VPS procedures per patient revealed that the vast majority (78.23%) underwent the procedure once, while the remaining patients had it performed twice, three times, or four times during the study period, accounting for 16.09%, 4.10%, and 1.58%, respectively. These patients received treatment across various hospitals in Saudi Arabia. The majority (59.99%) were treated in the Riyadh at KFH and KASCH hospitals, followed by 34.38% at KKH in Jeddah, with the remainder at smaller facilities in other cities (Table 2).

The clinical diagnosis of hydrocephalus among study participants is detailed in Table 3. The majority of patients (44.16%) did not have a specified class or etiology in the database. However, the class of hydrocephalus was specified in 26.50% of patients, with 42 patients diagnosed with obstructive hydrocephalus, 24 with communicating hydrocephalus, and 18 with normal-pressure hydrocephalus, accounting for 13.25%, 7.57%, and 5.68%, respectively. Conversely, the identified etiological factors of hydrocephalus were either congenital, as seen in 22 (6.94%) of study patients, including those with spina bifida, or acquired causes in 75 (23.66%) patients, including tumors, trauma, hemorrhage, infections, and other diseases.

### 3.2. Rate of VPS Infections

As detailed in Table 4, the prevalence of post-VPS infections was low during the study period, with no infections reported in 92.74% (294 patients). The remaining patients experienced infections at varying frequencies: 5.05% (16 patients) had one onset of infection, 1.89% (6 patients) had two, and 0.32% (1 patient) experienced three infections. The interval from VPS insertion to infection identification varied, with 35.48% of infections occurring within the first three days, suggesting an early onset for the majority of infections. Infections identified within two weeks post-procedure accounted for 58.06% of all cases, indicating a critical period for monitoring postoperative complications. Infections declined notably after the second week post-VPS surgery, with 9.68% occurring between two to four weeks and only 32.25% documented after one month.

Infection rates varied significantly across hospitals, with the highest occurrences reported at KKH in Jeddah, accounting for 47.83% of infections. In comparison, two hospitals in Riyadh, KFH and KASCH, collectively reported 52.18% of infections. On the other hand, no infections were reported from KAH in Al Ahsa, IABFH in Dammam, or PMBAH in Madinah during the study period. The causative agents of these infections were predominantly bacterial (93.55%), with a smaller fraction caused by fungal pathogens (6.45%) (Table 4).

### 3.3. Pathogens and Antibiotic Sensitivity Profiles 

**Figure 1:**
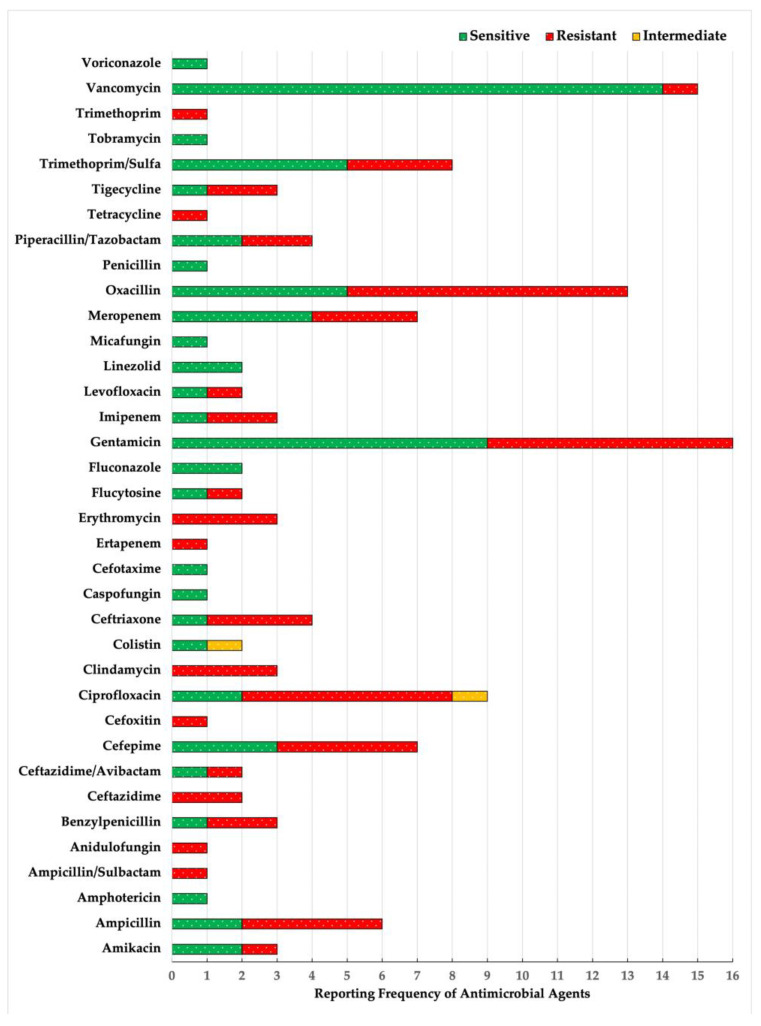
Reported patterns of antimicrobial efficacy and resistance among the isolated pathogens.

This study evaluated the infection rates and antibiotic sensitivity profiles across various pathogens isolated from patients following their VPS placement (Table 5, Figure 1). Coagulase-negative *staphylococci* (CoNS) were the most frequently isolated pathogens, detected on eight occasions (25.81%), with six of the strains being methicillin-resistant. *Staphylococcus aureus* was the second most common isolated pathogen, found in four instances (12.90%); three isolates were methicillin-sensitive, and one was resistant. Notably, all staphylococcal isolates demonstrated high susceptibility to vancomycin.

*Klebsiella pneumoniae* and *Escherichia coli* each accounted for 6.45% of the infections, exhibiting varied resistance patterns. For instance, one *K. pneumoniae* isolate was highly resistant to almost all tested antibiotics (16 out of 17 antibiotics), except for Ceftazidime/Avibactam. Conversely, *E. coli* was generally susceptible to piperacillin/tazobactam, with one strain also sensitive to ciprofloxacin (Table 5).

Other less frequently isolated organisms that pose significant concerns include *Pseudomonas aeruginosa*, *Acinetobacter ursingii*, and *Enterococcus faecium*, each associated with antibiotic resistance challenges. Noteworthy fungal pathogens such as *Candida parapsilosis* and *Cryptococcus neoformans* were also identified, with *Cryptococcus* demonstrating resistance to flucytosine but sensitivity to fluconazole and voriconazole (Table 5).

### 3.4. Clinical Outcomes

**Figure 2:**
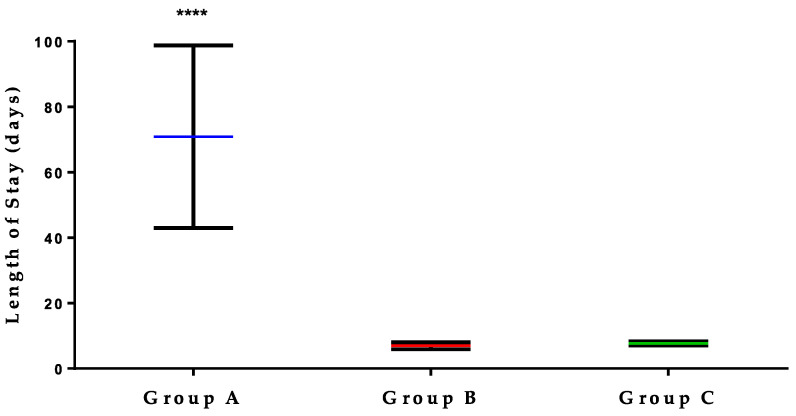
Impact of VPS infections on patient hospital stay duration. Patients were grouped by the presence of VPS infections during their stay. Group A represents the LOS of patients admitted with a VPS infection. Group B represents the LOS of the same patients in group A but during other admissions without a VPS infection. Group C represents the LOS of patients who were never diagnosed with a VPS infection during their hospital stay. Statistical analyses were conducted using one-way ANOVA followed by Dunnett’s multiple comparison test. There were statistically significant deviations observed in test group (A) compared to control groups (B) and (C), **** as indicated by a *p*-value < 0.0001.

The clinical impact of VPS infections was assessed by measuring the LOS as a primary indicator of morbidity. Figure 2 illustrates significant differences in LOS among patients with a VPS infection at the time of their admissions (Group A) compared to two control groups. The first control group includes the LOS of the same patients but during other hospitalization periods in which no infections occurred (Group B), and the second comprises patients who never experienced a VPS infection during any admission (Group C). Statistical analysis revealed that the mean LOS was substantially longer for Group A, averaging 70.84 days with considerable variance (SD = 139.5, median = 28). In contrast, Groups B and C, which included patients admitted without any infections, exhibited significantly shorter and more consistent LOS, with mean durations of 6.95 (SD = 11.09, median = 3) and 7.69 days (SD = 20.72, median = 2), respectively.

## 4. Discussion

Understanding hydrocephalus is crucial because it can lead to significant neurological impairment and life-threatening complications. The disease has multiple classification systems due to its complexity and the diverse etiological factors, many of which are unknown in a significant number of patients, as noted in our study (Table 3) [25,26]. Hydrocephalus is primarily classified into obstructive and communicating types, each of which can be further divided into congenital and acquired forms. Obstructive hydrocephalus occurs when the flow of the CSF is blocked between the brain’s ventricles, causing one or more ventricles to enlarge due to fluid buildup. In contrast, communicating hydrocephalus involves increased CSF levels due to abnormalities in CSF production or impaired drainage of excess fluid [25]. However, early diagnosis and management are essential to prevent irreversible damage and improve the quality of life for affected individuals [25,26].

VPS is commonly used to treat hydrocephalus, but associated infections are frequent and significantly increase morbidity, mortality, and healthcare costs [9,11,12]. Research on this topic in the Middle East, especially in Saudi Arabia, is limited, with inconsistent results [9,27,28,29,30,31]. These studies are typically limited by critical drawbacks, such as small sample sizes or their confinement to single-center observations. Additionally, many of these studies broadly evaluate VPS malfunctions without specifically addressing the types of infectious pathogens involved or examining their clinical impacts. Therefore, comprehensive multi-center research is essential to minimize this knowledge gap and establish solid, evidence-based protocols for managing VPS infections. Furthermore, such research could elucidate regional variations in infection rates and management efficacy, enabling healthcare systems to tailor interventions that are more effective at the national level and extend valuable knowledge to the international medical community.

In this study, 52.05% of the overall cohort were observed to be underweight, with a BMI less than 18.5. This finding aligns with the clinical challenges faced by hydrocephalus patients, which often result in poor appetite and nutritional issues [32]. Importantly, however, this prevalence increased significantly to 73% among those who developed VPS infections, highlighting a link between lower BMI and the incidence of postoperative infections. Numerous studies have demonstrated a clear correlation between malnutrition and an increased risk of postoperative infections across various types of surgical procedures, including neurosurgical procedures [33,34,35,36,37,38]. These findings underscore the critical need for comprehensive nutritional assessments and interventions as integral components of preoperative care for hydrocephalus patients. 

Although VPS effectively reduces the complications associated with hydrocephalus, it is not without risks, as shunt malfunctions are common and can lead to serious, sometimes life-threatening complications for patients [9]. Prior studies have documented VPS failure rates ranging from 17% to 33% [39,40,41,42,43,44]. In this study, approximately one in five patients (21.76%) experienced at least one VPS failure during the study period. This rate is within the expected range and aligns closely with findings from a large-scale study by Merkler et al. (2017), who reported that 23.8% of over 17,000 patients who underwent VPS suffered at least one complication over an average follow-up of 3.9 years (±1.8) [45]. 

Our data reveal that 29.52% of shunt failures were due to post-VPS infections. This incidence is comparable to a local study conducted by Alomar et al. (2023) which found a complication rate of 33.7% among shunted patients, with 11.42% from these malfunctions attributable to infections [29]. Remarkably, among the 23 patients who experienced post-VPS infections, recurrent infections were observed, occurring twice in 6 patients (26.08%) and three times in 1 patient (4.35%). Similar results have been seen in the meta-analysis study conducted by Freire-Archer et al. (2023), which reported a recurrence rate of 25% for deep surgical site infections after instrumented spinal fusion [46]. Additionally, surgical site infections typically follow a specific temporal pattern [47,48]. Previous investigations have shown that around two-thirds of infections related to VPS or external ventricular drains (EVDs) occur within the first month after surgery, with the rate increasing to as high as 90% within the first year post-surgery [49,50,51,52,53]. Consistent with these findings, we found that more than half of the infections were reported within the first two weeks following the VPS procedure, with over 80% of infections reported within the first year. This underlines the importance of intensified monitoring and immediate treatment actions in the first month after surgery to effectively reduce the development of infections and their consequences for patients.

VPS infections are frequent post-surgery complications that arise from the contamination of either the surgical wound or implanted medical devices [52]. The sources of contamination include microorganisms acquired from the hospital environment or the community, as well as various opportunistic microbes already present within the body [54,55]. In this study, Gram-positive bacteria were found to be the leading cause of infections (58.08%), followed by Gram-negative bacteria and fungal agents (35.46% and 6.46%, respectively). Within those isolates, *Staphylococci* emerged as the main cause of infections in shunted patients, accounting for 38.71% of total infections. Of the staphylococcal infections, two-thirds were caused by skin flora CoNS, and the remaining infections were caused by *S. aureus*. These results align with a substantial body of prior research from local, African, and Western studies which consistently attribute CSF diversion devices infections to Gram-positive pathogens, predominantly *Staphylococcal* species. Conversely, the remaining infections were due to Gram-negative organisms at variable rates [28,49,56,57,58]. In a marked contrast, a study conducted in Pakistan documented an exceptionally high incidence of infections caused by Gram-negative bacteria, accounting for 76% of VPS-related infections. Notably, infections attributed to *Acinetobacter* species alone represented 41% of these cases [15]. However, we believe that discrepancies in study design and variations in infection reporting practices across different countries and hospitals could explain such divergent findings. 

It is also important to note that rare pathogens have been isolated from shunted patients in our study. In fact, this is not surprising since patients with hydrocephalus are considered immunocompromised and are more prone to infection by low-risk opportunistic organisms [59,60,61]. Moreover, although a significant proportion of the isolated pathogens demonstrate a pronounced susceptibility to antibiotics, a subset shows significant resistance, presenting challenges to effective therapeutic interventions. Interestingly, the majority of the *S. aureus* isolates demonstrated sensitivity to methicillin, with only one isolate exhibiting resistance. In contrast, a substantial proportion of CoNS isolates (six out of eight) displayed resistance to methicillin. Nonetheless, vancomycin was found to be effective against all *Staphylococcal* species, indicating its continued efficacy and reliability as a treatment option for such infections. This is supported by the findings of Asif et al., who documented that all Staphylococcal isolates from VPS infections in their study (n = 2580) were sensitive to vancomycin. However, in a different outcome from ours, they also revealed a significant presence of methicillin-resistant *S. aureus* (MRSA), with nearly half of the *S. aureus* isolates (719/1343) exhibiting resistance [15]. 

We also reported three MDR isolates with high levels of resistance, including *K. pneumoniae*, *P. aeruginosa*, and *A. ursingii*. Remarkably, the World Health Organization has classified the first two isolates as priority pathogens, directing research and development efforts towards combating antimicrobial resistance. Although the last isolate, *A. ursingii*, is not specifically classified due to its rarer incidence, it remains a challenging bacterium to manage, as it belongs to the same genus as the notoriously resistant *Acinetobacter baumannii* [62]. Given this challenge, regular assessments and refinements of prophylactic antibiotic protocols are important to minimize the risk of VPS failure due to MDR infections. 

The duration of a patient’s hospital stay, or LOS, is a critical indicator of morbidity and a crucial determinant of healthcare costs [63,64]. It is well documented that postoperative infections significantly prolong LOS, although the extent of this prolongation varies across studies due to differences in infection sites and patient health conditions [65,66,67,68]. A recent large-scale study involving nearly 4.5 million patients showed that postoperative complications, including surgical site infections, which accounted for 1.0%, are associated with a significant increase in LOS, ranging from 3.9 to 20.1 days [65]. In this report, we observed a median LOS of 28 days, similar to findings by Conen et al., who reported a median hospitalization of 26 days among patients with VPS infections [49]. This figure is approximately 10 times higher than the normal LOS, which underscores the importance of improving postoperative care to reduce LOS and associated healthcare costs.

Healthcare-associated infections (HAIs) are a major global health concern, associated with increased morbidity and mortality among patients [69]. Alarmingly, up to 50% of these infections involve pathogens that exhibit MDR [70,71]. Moreover, within the Middle East, the incidence and diversity of pathogens causing HAIs differ significantly among countries and the various healthcare centers [72,73,74]. Notably, this study highlights a significant discrepancy among hospitals, as approximately half of the VPS infections were documented at KKH in Jeddah, despite it serving only 34.38% of the study cohort. In contrast, the remaining infections were detected in hospitals in Riyadh, which account for 58.99% of the study patients, while no cases were reported in the other MNGHA hospitals. 

Studies have shown that differences in healthcare settings, hospital types and characteristics, and patient demographics are potential factors contributing to the variability in the prevalence of HAIs observed across various countries and regions [73,75,76,77]. A key factor could be variations in empirical treatment protocols and monitoring systems among hospitals. Additionally, surgical practices could significantly impact infection rates due to variations in health workers’ experience, adherence to surgical guidelines, and the complexity of treated cases. The infrastructure and layout of the hospital, which affect how well infection control practices are enforced, might also play a crucial role. Another critical aspect to consider is patient demographics, since the patient population at KKH may have unique characteristics that predispose them to higher risks of infection. These could include a higher prevalence of comorbidities, differences in socioeconomic status, or variations in access to healthcare prior to receiving surgical intervention.

To address these issues, it is crucial for MNGHA to conduct a thorough review of infection control practices and resource distribution to effectively manage the elevated infection rates at specific hospitals. This review should include an evaluation of the surgical and hygiene protocols at KKH in comparison to other centers, as well as an analysis of patient demographics and preoperative health conditions. Such a comprehensive approach will help identify targeted interventions that could reduce the risk of HAIs and improve patient outcomes.

To our knowledge, this is the first study analyzing data from multiple hospitals in Saudi Arabia, and possibly the broader Middle East, investigating infection rates, causative pathogens, and related outcomes in VPS patients. Its extensive regional coverage and considerable sample size underscore its significance in understanding healthcare disparities. The findings are expected to standardize and coordinate healthcare practices, thereby enhancing regional management of healthcare challenges. However, this study’s retrospective nature limited the depth of temporal analysis, which affected our ability to fully explore the impact of VPS infections and assess additional outcomes, suggesting this as a future area for investigation. Moreover, restricted access to complete patient reports impeded the evaluation of specific interventions used to manage infections and other health conditions affecting shunted patients. This also limited our ability to assess the morbidity levels in patients with VPS infections, with length of stay being the only indicator used. Nonetheless, despite these limitations, this study provides critical insights and serves as a valuable benchmark for future research, potentially leading to more integrated strategies for managing regional healthcare challenges and making a significant contribution to the existing body of knowledge.

## 5. Conclusions

This study highlights the essential need for robust infection control measures and the standardization of clinical protocols in the management of VPS for hydrocephalus. Our findings demonstrate that while VPS effectively improve patient outcomes, postoperative infections pose substantial challenges, leading to increased morbidity, prolonged hospital stays, and consequently elevated healthcare costs. The variability in infection rates across different hospitals suggests that localized factors could significantly influence the risk and outcomes of these infections. Facilities with high infection rates should reassess their infection control practices and consider integrating tailored health and nutritional support to enhance patient care. Furthermore, the prevalence of MDR pathogens calls for comprehensive antimicrobial stewardship to minimize the risk of empirical treatment failure. Therefore, it is crucial for healthcare facilities to adopt evidence-based strategies to reduce the incidence of VPS-related infections and to implement robust monitoring systems that rapidly identify and reduce the potential risks. Addressing these challenges will not only enhance the safety and effectiveness of hydrocephalus treatments but also alleviate the burden on healthcare systems and substantially improve the quality of life for affected patients. Additionally, the insights gained from this study could help to refine treatment protocols in Saudi Arabia and offer valuable contributions to the global medical community.

## Figures and Tables

**Table 1:** List of hospitals operated by the Ministry of National Guard Health Affairs (MNGHA) in Saudi Arabia [24].

Hospital	Region	City	Capacity
King Fahad Hospital (KFH)	Central	Riyadh	1973 beds
King Abdullah Specialist Children Hospital (KASCH)	Central	Riyadh	346 beds
King Khalid Hospital (KKH)	Western	Jeddah	756 beds
Prince Mohammed Bin Abdulaziz Hospital (PMBAH)	Western	Madinah	215 beds
King Abdulaziz Hospital (KAH)	Eastern	Al Ahsa	300–400 beds
Imam Abdulrahman Bin Faisal Hospital (IABFH)	Eastern	Dammam	108 beds

**Table 2:** Summary of the study cohort demographics.

	Demographic Data	Frequency (*N* = 317)	Percentage
**Sex**	Male	151	47.63%
	Female	166	52.36%
**Average BMI**	<18.5	165	52.05%
	18.5–24.9	53	16.72%
	25–29.9	41	12.93%
	30–34.9	34	10.73%
	≥35	11	3.47%
	N/A	13	4.10%
**Birth condition**	Full term (38–42 weeks)	31	9.78%
	Pre-term (≤37 week)	43	13.56%
	Not specified	243	76.66%
**Age at 1^st^ VPS Procedure**	0–4 weeks	29	9.15%
	1–2 months	28	8.83%
	3–6 months	30	9.46%
	7–12 months	11	3.47%
	1–2 years	33	10.41%
	3–16 years	74	23.34%
	17–30 years	21	6.62%
	31–65 years	60	18.93%
	>65 years	31	9.78%
**Frequency of VPS Procedures**	1	248	78.23%
	2	51	16.09%
	3	13	4.10%
	4	5	1.58%
**Hospital**	KFH, Riyadh	133	41.96%
	KASCH, Riyadh	54	17.03%
	KKH, Jeddah	109	34.38%
	KAH, Al Ahsa	13	4.10%
	IABFH, Dammam	4	1.26%
	PMBAH, Madinah	4	1.26%

**Table 3:** Distribution of hydrocephalus classes and etiology among the study cohort.

Clinical Diagnosis ^1^	Frequency (*N* = 317)	Percentage
Communicating hydrocephalus, unspecified etiology	24	7.57%
Congenital hydrocephalus, unspecified class	18	5.68%
Hydrocephalus associated with other diseases, unspecified class	22	6.94%
Hydrocephalus in neoplastic disease, unspecified class	31	9.78%
Normal pressure hydrocephalus, unspecified etiology	18	5.68%
Obstructive hydrocephalus, spina bifida myelomeningocele/meningocele	4	1.26%
Obstructive hydrocephalus, unspecified etiology	38	11.99%
Post-traumatic hydrocephalus, unspecified class	4	1.26%
Post-hemorrhage hydrocephalus, unspecified class	11	3.47%
Postinfectious hydrocephalus, unspecified class	7	2.21%
Non-specified class/etiology	140	44.16%

^1^ The ‘Non-specified class/etiology’ category includes cases where the patient’s medical records did not document the specific class or cause of hydrocephalus.

**Table 4:** Summary of infection data.

Variable		Frequency (Total Number)	Percentage
**Number of post-VPS infections per patient**	0	294 (317)	92.74%
	1	16 (317)	5.05%
	2	6 (317)	1.89%
	3	1 (317)	0.32%
**Post-VPS infection interval**	1–3 days	11 (31)	35.48%
	4–14 days	7 (31)	22.58%
	2–4 weeks	3 (31)	9.68%
	1–3 months	4 (31)	12.90%
	>3 months	6 (31)	19.35%
**Average BMI of patients who experience post-VPS infections**	<18.5	17 (23)	73.91%
	18.5–24.9	3 (23)	13.04%
	25–29.9	1 (23)	4.35%
	30–34.9	1 (23)	4.35%
	≥35	0 (23)	0.00%
	N/A	1 (23)	4.35%
**Rates of patients who experience post-VPS infections by hospital**	KFH, Riyadh	6 (23)	26.09%
	KASCH, Riyadh	6 (23)	26.09%
	KKH, Jeddah	11 (23)	47.83%
	KAH, Al Ahsa	0 (23)	0.00%
	IABFH, Dammam	0 (23)	0.00%
	PMBAH, Madinah	0 (23)	0.00%
**Causative Agents**	Bacterial	29 (31)	93.55%
	Fungal	2 (31)	6.45%

**Table 5:** Infection rates and antimicrobial susceptibility profile of reported pathogens.

Pathogen	Infection Rates	Susceptibility Findings ^1^
Coagulase-negative *Staphylococcus*	8 (25.81%)	Van (S), Tmp/Sul (S), Oxa (R)
		Van (S), Oxa (S)
		Van (S), Oxa (R)
		Van (S), Tet (R), Tmp/Sul (R), Oxa (R), Gen (R), Ery (R), Cli (R), Cipf (R)
		Van (S), Oxa (S), Ben (R)
		Van (S), Oxa (R), Gen (R), Ery (R), Cli (R), Cipf (R)
		Van (S), Trimth (R), Oxa (R)
		Van (S), Oxa (R), Gen (R), Ery (R), Cli (R), Cipf (R)
*Staphylococcus aureus*	4 (12.90%)	Van (S), Oxa (S)
		Van (S), Oxa (S)
		Van (S), Tmp/Sul (S), Oxa (R), Lin (S)
		Van (S), Oxa (S), Gen (S)
*Klebsiella pneumoniae*	2 (6.45%)	Tmp/Sul (S), Pip/Taz (S), Gen (S), Cipf (I), Cftx (R), Amp (R)
		Amk (R), Tmp/Sul (R), Pip/Taz (R), Mer (R), Imp (R), Gen (R), Cipf (R), Cftx (R), Ceaz (R), Cfox (R), Cefp (R), Amp (R), Gen (R), Ert (R), Tgc (R), Cefp (R), Ceaz/Avi (S)
*Escherichia coli*	2 (6.45%)	Tmp/Sul (S), Pip/Taz (S), Gen (S), Cipf (S), Cftx (S)
		Amk (S), Tmp/Sul (R), Mer (S), Imp (S), Gen (S), Cipf (R), Cftx (R), Amp (R), Tgc (S), Col (I)
*Enterococcus faecalis*	2 (6.45%)	Amp (S), Gen (S)
		Amp (S), Gen (S)
*Enterobacter cloacae*	2 (6.45%)	Mer (S), Gen (S), Cefp (S), Gen (R)
		Mer (S), Cefp (S), Gen (R)
*Pseudomonas aeruginosa*	1 (3.23%)	Amk (S), Tob (S), Pip/Taz (R), Mer (R), Imp (R), Gen (S), Cipf (R), Ceaz (R), Col (S), Ceaz/Avi (S), Cefp (R), Lev (R), Tgc (R)
*Acinetobacter ursingii*	1 (3.23%)	Mer (R), Cef (R), Amp/Sul (R)
*Enterococcus faecium*	1 (3.23%)	Van (R), Amp (R), Lin (S)
*Micrococcus luteus*	1 (3.23%)	Van (S), Oxa (R), Ben (R)
*Serratia marcescens*	1 (3.23%)	Mer (S), Gen (S), Cipf (S), Cefp (S)
*Stenotrophomonas maltophilia*	1 (3.23%)	Tmp/Sul (S), Lev (S)
*Streptococcus viridans* group	1 (3.23%)	Van (S), Pen (S), Ctx (S)
*Bacillus* species	1 (3.23%)	Ben (S)
Mix growth of *Moraxella catarrhalis* and *Lactococcus garvieae*	1 (3.23%)	N/A
*Candida parapsilosis*	1 (3.23%)	Flc (S), Cspf (S), Mcf (S), Flz (S)
*Cryptococcus neoformans*	1 (3.23%)	Amph (S), Flc (R), Anf (R), Vor (S), Flz (S)

^1^ Amikacin (Amk), Ampicillin (Amp), Amphotericin (Amph), Ampicillin/Sulbactam (Amp/Sul), Anidulofungin (Anf), Benzylpenicillin (Ben), Ceftazidime (Ceaz), Ceftazidime/Avibactam (Ceaz/Avi), Cefepime (Cefp), Cefoxitin (Cfox), ciprofloxacin (Cipf), Clindamycin (Cli), Colistin (Col), Ceftriaxone (Cftx), Caspofungin (Cspf), Cefotaxime (Ctx), Ertapenem (Ert), Erythromycin (Ery), flucytosine (Flc), fluconazole (Flz), Gentamicin (Gen), Imipenem (Imp), Levofloxacin (Lev), Linezolid (Lin), Micafungin (Mcf), Meropenem (Mer), Oxacillin (Oxa), piperacillin/tazobactam (Pip/Taz), Tetracycline (Tet), Tigecycline (Tgc), Trimethoprim/Sulfa (Tmp/Sul), Tobramycin (Tob), Trimethoprim (Trimth), vancomycin (Van), voriconazole (Vor).

## Data Availability

The data presented in this study are available from the corresponding author upon reasonable request. The data are not publicly available due to restrictions in data privacy.
